# Length and activity of the root apical meristem revealed *in vivo* by infrared imaging

**DOI:** 10.1093/jxb/eru488

**Published:** 2014-12-24

**Authors:** François Bizet, Irène Hummel, Marie-Béatrice Bogeat-Triboulot

**Affiliations:** ^1^INRA, UMR Ecologie et Ecophysiologie Forestière,F-25420 Champenoux, France; ^2^Université de Lorraine, UMR Ecologie et Ecophysiologie Forestière, BP 239, F-54506 Vandoeuvre, France

**Keywords:** Cell division rate, infrared imaging, kinematics, osmotic stress, poplar, root apical meristem.

## Abstract

Understanding how cell division and cell elongation influence organ growth and development is a long-standing issue in plant biology. In plant roots, most of the cell divisions occur in a short and specialized region, the root apical meristem (RAM). Although RAM activity has been suggested to be of high importance to understand how roots grow and how the cell cycle is regulated, few experimental and numeric data are currently available. The characterization of the RAM is difficult and essentially based upon cell length measurements through destructive and time-consuming microscopy approaches. Here, a new non-invasive method is described that couples infrared light imaging and kinematic analyses and that allows *in vivo* measurements of the RAM length. This study provides a detailed description of the RAM activity, especially in terms of cell flux and cell division rate. We focused on roots of hydroponic grown poplars and confirmed our method on maize roots. How the RAM affects root growth rate is studied by taking advantage of the high inter-individual variability of poplar root growth. An osmotic stress was applied and did not significantly affect the RAM length, highlighting its homeostasis in short to middle-term responses. The methodology described here simplifies a lot experimental procedures, allows an increase in the number of individuals that can be taken into account in experiments, and means new experiments can be formulated that allow temporal monitoring of the RAM length.

## Summary

A fast and *in vivo* approach to assess the dynamics of root apical meristem length.

## Introduction

Root growth takes place in the root apex, nesting both cell division and cell expansion. As these cellular processes are both time and space separated, the zonation patterns of a root apex are classically separated into a cell division zone and an elongation zone. In such patterns, the cell division zone could be defined as the root apical meristem (RAM), producing cells that are progressively pushed into the elongation zone where they stop dividing and start to rapidly increase in length. Nevertheless, the spatial demarcation between the cell division zone and the cell expansion zone in a root is not straightforward, complicating the physiological characterization of the root apex. Some proposed a transition zone between, or overlapping, the cell division zone and the elongation zone, others divided the RAM into a proximal and a basal meristem (see Ivanov and Dubrovsky, 2013 for review). For clarity, here, the terminology recently proposed by Ivanov and Dubrovsky (2013) is adopted, where the RAM includes both a cell proliferation domain where cells maintain a high probability to divide and a transition domain with a low probability of cell division occurrences.

As proposed by Beemster *et al.* (2003), root growth is an integrative process depending on whole-organism signalling and individual growth trajectories of cells. The number of dividing cells in the RAM generates a cell flux reported to be of importance in modulating root growth (Baskin, 2013). From the continuity equation, the cell flux is the integral of the cell production rate along the RAM, which depends on the number of dividing cells and on cell division rate (Erickson and Silk, 1980). Studies of cell length profiles provided evidence that the proliferative fraction (i.e. the fraction of dividing cells) in the RAM proliferation domain is indistinguishable from one, even in response to moderate levels of stress (Baskin, 2000; Ivanov *et al.*, 2002). Thus, owing to the constancy of cell length in the cell proliferation domain, the RAM length is a key indicator of the number of dividing cells (Ivanov, 1997; Beemster and Baskin, 1998). Concerning cell division rate, there are still debates about its constancy along the RAM. Although some studies suggest that previously reported non-constant rates could result from methodological bias such as over smoothing on few data-points (Beemster and Baskin, 1998; Baskin, 2000), there is still a lack of experimental data demonstrating this constancy.

From a physiological point of view, the RAM transition domain is a site of integration of hormonal signals including auxin and cytokinin (Dello Ioio *et al.*, 2007; Perilli *et al.*, 2012), but also gibberellins (Achard *et al.*, 2009), brassinosteroids (Gudesblat and Russinova, 2011), and ethylene (Skirycz *et al.*, 2011). Reactive oxygen species were also identified as key components determining the transition between cell proliferation and cell elongation (Tsukagoshi *et al.*, 2010). Physiological variations of the concentration of such compounds result in variation of the RAM length. Unfortunately, focusing on the RAM is problematic, as even its length measurement depends on the authors’ conception of what belongs or not to the RAM (Ivanov and Dubrovsky, 2013). Indeed, although the most apical limit of the RAM is well defined, starting at the quiescent centre, its shootward border is often determined in a variable way on the basis of increasing cortical cell length. The cortical cell length threshold that determines the end of the RAM is arbitrarily set either qualitatively (from direct cell observation) or quantitatively (from cell length measurement) but often without providing the adopted threshold values (see Casamitjana-Martınez *et al.*, 2003; Dello Ioio *et al.*, 2007; Achard *et al.*, 2009; Ubeda-Tomás *et al.*, 2009; Hacham *et al.*, 2011; Makkena and Lamb, 2013; Peng *et al.*, 2013 for examples). Apart from the subjective aspect of such a methodology, the use of a threshold in cell length has been shown to provide good accuracy for estimating the number of cells within the RAM (Beemster, 2002). It should be noted that most analyses of the RAM were done for *Arabidopsis*, a species exhibiting few cortical cell layers. Setting the RAM shootward border for other species is far more time-consuming, owing to numerous cortical layers and longer RAM.

In this study, a new non-destructive methodology is presented to determine the length of the RAM from *in vivo* near infrared imaging. This simple method is shown to provide accurate values of the RAM length. Considering a large panel of poplar roots with various growth rates, the method was validated by comparing the obtained RAM length with classical cell length measurements. A range of osmotic stresses was applied to analyse the short-term response of the RAM length to an environmental constraint. The analysis of the inter-individual activity of the RAM (cell division rate and elongation rate) provided cues for a deeper understanding of the link between RAM length and root growth.

## Materials and methods

### Growth conditions and application of the osmotic stress

Cuttings of a commercial hybrid poplar (*Populus deltoides* × *Populus nigra*, cv ‘Soligo’) were grown in hydroponics (half-strength Hoagland nutrient solution supplemented with 0.8mM KH_2_PO_4_ and adjusted to pH 5.8). The solution was bubbled to prevent hypoxia and renewed every 2–3 d. Buds were removed to prevent leaf appearance. The culture system was placed in a dark room (air temperature: 23.7±0.9°C; atmospheric humidity: 60±4%). Cuttings emitted adventive roots after approximately 10 d. Only one root per cutting was considered and randomly assigned to a modality of treatment. Growth analyses were performed over July and August 2012, independently on 28 roots.

Once an adventive root was longer than 2cm (about 3 d after root emergence), the cutting was transferred into a transparent Plexiglas^**®**^ tank filled with bubbled and circulating nutrient solution. Osmotic stress was applied by adding polyethylene glycol to the nutrient solution (PEG 4000, Merck chemicals). PEG concentrations of 100, 130, 160g l^–1^ were applied, generating, respectively, low (0.21MPa, *n*=6 roots), moderate (0.27MPa, *n*=10 roots) and high (0.37MPa, *n*=4 roots) osmotic stress levels determined using a vapour pressure osmometer (Wescor 5500). Osmotic pressure of the nutrient solution without PEG was 0.04MPa (*n*=8 roots). Osmotic stress was applied during the growth monitoring: the nutrient solution in the tank was replaced by a PEG-added nutrient solution by changing the incoming solution with a three ways faucet. It took about 3min for the PEG solution to totally replace the control solution without any manipulation of the root or pause in the growth monitoring. Oxygen level in solutions was measured using an oxymeter (HQ40D, Lange).

### Root growth monitoring and statistical analyses

Root growth was monitored with no visible light under near-infrared illumination (λ=850nm) with a LED lamp placed at about 15cm of the root apex (photon flux density: 0.5–1.5 µmol m^–2^ s^–1^). A black background coupled with a low incidence angle light generated natural marks on the root surface. Pictures were taken with a defiltered camera (Nikon D80). The camera was mounted with a macro objective (Nikkor 60mm) and 56mm of extension tubes (Kenko). Focus distance was set at minimal value (around 102.5mm) on the objective and focus was done using an optical rail on which the camera was set. For automation, the camera was computer-controlled using Camera Control Pro software (Nikon, v1.3). Shots were taken during at least 4–5h per root. Raw velocity profiles were obtained by particle image velocimetry, from the monitoring of mark displacement along the root apex, using Kineroot and the highest correlation coefficient search algorithm (Basu *et al.*, 2007, Matlab R2011b, v7.13.0.564). Depending on the root and on the time point, velocity profiles were either sigmoidal, bilinear, or a variable mix of both. A composite function established by Peters and Baskin (2006) was fitted, which gives more reliable fits than other growth equations (like Gompertz or Richard’s equations). The adjustments were done using R with the non-linear least square function (nls). Starting values were determined by simulated annealing. All statistical tests including variance analysis, Tukey’s tests, and linear adjustments were done considering an alpha risk of 0.05. The root mean square error (RMSE), i.e. the standard deviation of the differences between predicted and observed values, was computed to assess the quality of prediction. The lower the RMSE, the better the prediction.

### Infrared picture analysis

Illumination by near-infrared light generated a brightness gradient along the root apex that was used to determine the RAM length. Analysis of root brightness was done using Fiji (Schindelin *et al.*, 2012) on raw images after the application of a Gaussian blur filter (radius of 40–50 µm). Pixel intensity was measured on a thick segmented line traced along the root centre. The RAM length was determined for each root as being the distance between the quiescent centre and the first point where pixel intensity dropped below 75% of the maximal pixel intensity, pixel intensity in the mature zone being the offset.

### Histological analyses

After growth monitoring, all root apices were immediately fixed in a phosphate-buffered saline solution containing 3.5% paraformaldehyde and 0.5% glutaraldehyde under partial vacuum for 30min. Apices were then stored at 4 °C. Fixed samples were rinsed with distilled water and moved through an ethanol dehydration series: 30min into 30%, 50%, 70%, 95%, and 100% ethanol baths. Root apices were finally infiltrated and carefully embedded into a fast cold curing resin (Technovit^®^ 7100) following manufacturer’s instructions. Longitudinal sections of 5 µm were cut using a rotary microtome (Microm HM355S, Thermo Scientific) equipped with a tungsten carbide knife. Sections were coloured with toluidine blue and mounted with Eukitt^®^ mounting medium.

Pictures of longitudinal sections were taken under one hundred magnification (camera Leica DFC420C, Leica Microsystems) and assembled using a dedicated software (Autopano Giga, Kolor, v2.6.4). Pictures were analysed using Fiji (Schindelin *et al.*, 2012).Vacuolization was estimated from the colouration gradient along the root apex. For each root, vacuolization corresponded to the drop in relative pixel intensity below 50% of the maximal value. Cell length was measured semi-automatically as the distance between two consecutive transverse cell walls through the analysis of colour intensity (toluidine blue staining). The distance of a cell from the quiescent centre was defined as the midpoint of the cell reported on a segmented line passing along the centre of the root.

## Results and discussion

### A novel and non-destructive method to determine the length of the root apical meristem

Here, the length of the root apical meristem (RAM) was defined as the distance between the quiescent centre and a shootward border, settled according to a cortical cell length threshold (Casamitjana-Martınez *et al.*, 2003; Hacham *et al.*, 2011). This threshold separates small and proliferative cells from large fast-expanding cells. Given that 99% of cortical cells within the apical part of the cell proliferation domain (first 500 µm of the root, about 110 cells measured per root) were below 22.3 µm in length (Supplementary Fig. S1), this value was chosen as the threshold. For each cortical layer, the x-coordinate along the root axis of the first cell that was longer than the threshold (excluding the first 500 µm) was determined. At the whole-root scale, the RAM shootward border was computed as the mean x-coordinate for all cortical layers (Supplementary Fig. S2). By doing so, the same weight is given to each cortical cell layer in the determination of the RAM shootward border. The later could alternatively be positioned from the profile of mean cell length, but it would give more weight to files where cells still divide (generating two times more cells for the profile) and thus may overestimate the mean position of the arrest of cell division, especially for species with several layers of cortical cells (as in poplar or maize). The cell length threshold of 22.3 µm is lower than the 40–45 µm previously found in *Arabidopsis* (Beemster, 2002; West, 2004). The threshold was about three times the length of the smallest cortical cells (Supplementary Fig. S1) and a similar ratio has already been mentioned for *Arabidopsis* (Baskin *et al.*, 1995; Liu *et al.*, 2013).

**Fig. 1.**
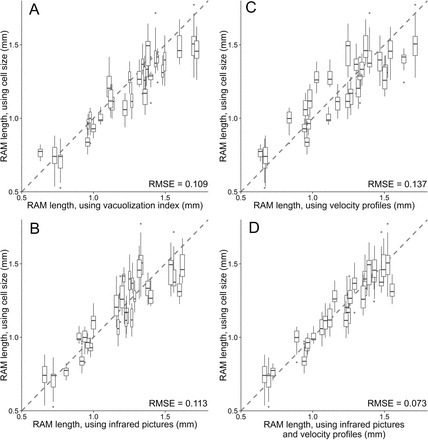
Comparisons of meristem length determined on the basis of cell length and other methodologies. Relationships are given for vacuolization index (A), brightness intensity in infrared pictures (B), c1 parameter values determined from adjustment of growth profile (C), and combination of those two methods (D) against cell length measurement. Boxplot in y-axis is the result of the lengths obtained in the different cell files. Dashed line=1:1 relationship. Root-mean-square errors (RMSE) are given for the 1:1 relationship.

As highlighted by Ivanov (1997), the marked rise in relative growth rate is the most valid criterion of the cell transition to elongation, rather than change in cell shape or mitosis cessation. An increase of the degree of cell vacuolization accompanies the increase in cell length, which is a strong indicator that the cell will not divide anymore (Achard *et al.*, 2009). At the whole-root scale, an increase in vacuolization index is expected at the limit between the RAM transition domain and the elongation zone, thus indicating the position of the RAM shootward border. As the vacuole enlarges, the cytosol is flattened on the cell wall. A longitudinal section of a root apex coloured with toluidine blue highlights the decrease of the colouration intensity as the vacuolization increases (Supplementary Fig. S3). The use of histological staining has not been employed yet to assess vacuolization intensity, although it has already been suggested (De Veylder *et al.*, 2001). The wide range of RAM length in the poplar dataset here revealed that the degree of vacuolization strongly correlated with the position of the RAM shootward border (RMSE=0.109, Fig. 1A). Estimation of the RAM length from coloured sections seems to be conclusive, precluding tedious cell length measurements.

**Fig. 2.**
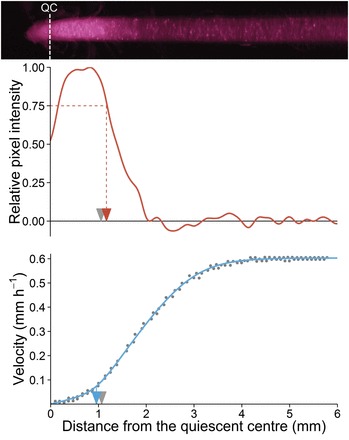
Determination of the RAM length from infrared pictures (red) and from velocity profiles (blue). Grey arrow indicates the meristem length determined from the cell length profile. The mean between the relative pixel intensity profiles (red arrow) and the first inflexion point of the velocity profile (blue arrow) gave the best estimate of the meristem length (see figure 1). QC: Quiescent centre.

Given that the histological approach is time consuming and restrains data only to an end-point snapshot, new tools were developed enabling a non-destructive measurement of the RAM length and allowing thus the monitoring of its temporal variation. Two possibilities were considered: (i) using directly a raw infrared picture of the root apex or (ii) using the velocity profile computed from kinematic analysis. In biological material, speckle patterns are commonly observed in response to laser light and has been shown to co-localize with a thigmostimuli responsive zone in roots (Ribeiro *et al.*, 2014). One hypothesis is that the near-infrared light used for growth monitoring could be differentially reflected depending on various components such as cell wall orientation and cell density. Under infrared illumination, a systematic zone of high brightness occurred in the most apical part of the root and could be measured. Using brightness intensity as an indicator of cell wall density and thus of the RAM length was tested. Concerning the kinematic approach, a parameter of the growth equation used for the adjustment of velocity profiles was considered. This parameter *c1* represents the transition point between the first and the second linear domain of the velocity profile (for details see Peters and Baskin, 2006). Such an inflexion point should be a good indicator of the position where cell length starts to rapidly increase and has already been used for characterizing the RAM length (Ma, 2003; Wuyts *et al.*, 2011). The RAM length obtained from cell length profiles, considered as the closest proxy of the RAM length, was compared to the length of infrared brightness zone and to the value of *c1*. The infrared brightness was a good estimator of the RAM length (RMSE=0.113, Fig. 1B), as the *c1* parameter (RMSE=0.137, Fig. 1C). A 1:1 relationship was found between these two parameters (RMSE=0.205), indicating that both estimators were highly congruent even if underlying features were distinct. Finally, a mathematical combination of these two estimators (i.e. the average) gave the most reliable proxy of the RAM length computed from cell length (RMSE=0.073, Fig. 1D). For poplar, the threshold of infrared brightness was set to 75 % of maximal intensity, other thresholds (ranking from 60 to 90%) providing strong linear relationships with the RAM length although not a 1:1 relationship. For subsequent analyses, this combination of infrared brightness and *c1* values was kept to determine the RAM length (summarized in Fig. 2).

The procedure was cross validated for non-destructive determination of the RAM length by testing its reliability in others species. On five hydroponic-grown maize roots, a highly significant correlation was found between the RAM length obtained from cell length profiles and from *in vivo* assessment. A disconnect from 1:1 relationship was found, meaning that even if the methodology gives reliable relative data between individuals, a calibration step is required to obtain absolute values of the RAM length through linear regression (Supplementary Fig. S4). The suitability of infrared light to assess RAM length was checked on 20 roots of Petri dish-grown *Arabidopsis* plants (Supplementary Fig. S5). Calibration of infrared threshold to 85% of maximal pixel intensity gave a confident 1:1 relationship (RMSE=0.038), validating the procedure in fine roots.

*In vivo* measurements of the RAM length offer several advantages. By skirting around a methodological constraint (the microscopy protocol), this methodology allows (i) increasing the number of individuals that can be taken into account in an experiment, (ii) monitoring the temporal variation of the RAM length in response to many cues, (iii) opening the RAM length measurements to new fields (e.g. it could be used to estimate the RAM activity in field rhizotrons, Pagès *et al.*, 2010), and (iv) avoiding potential artefacts induced by manipulation during sample preparation (e.g. shrinking during fixation).

### What does the measured RAM shootward border correspond to?

As previously defined, the RAM includes a transition domain where both division and elongation occur. Although in the method described here the RAM shootward border agreed well with cell length profiles, information about cell processes are required to ensure that the RAM shootward border coincide with the end of the RAM transition domain. To further address this question, both cell flux and cell division rate were characterized, before and after this shootward border. Assuming the steady state of root growth, the cell flux was determined as the ratio between velocity and cell length (Fiorani and Beemster, 2006). Cell flux at the RAM shootward border (4.8±0.4 cell h^–1^) was similar to that measured at the end of the growth zone (5.1±1.1 cell h^–1^), confirming that cells already stopped dividing at the RAM shootward border. Meanwhile, elongation rates at the RAM shootward border were low (mean=0.09mm h^–1^) and highly variable between individuals (standard deviation=0.02mm h^–1^); thus, a few late divisions could be masked.

**Fig. 3.**
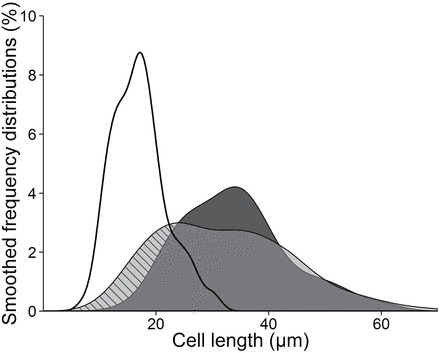
Smoothed frequency distribution of cell length. Solid line refers to an initial cell population located at the RAM shootward border. The light grey zone corresponds to the measured distribution of cell length at the expected location after two hours of expansion. The dark grey zone corresponds to the expected distribution of cell length at the same location if no division occurred for the two hours. Hatchings indicate cells smaller than expected without division.

To increase the accuracy of the measurements, the high cell density on longitudinal sections was used to analyse cell populations at the RAM shootward border and beyond. As microscopy provides only an end-point measurement that precludes direct analysis of time-related effect, steady growth and stability of cell length profiles along time were assumed. Temporal monitoring of root growth was used to estimate the displacement of a cell population initially located at the RAM shootward border. After two hours of root growth, the length of the cells within this location were measured. Then, hypothesizing that cells located at the RAM shootward border do not divide anymore, their expected length was calculated from their measured elongation rate during two hours of growth. The expected distribution of cell length was compared to the measured one (Fig. 3). A good correspondence between cell length distributions in expected and measured cell populations was found for long cells, confirming the temporal stability of root growth. When focusing on the shortest cells, measured lengths were shorter than expected without divisions (see hatchings in Fig. 3), suggesting that some cells divided after exiting the RAM shootward border where vacuolization already started, as already underlined by Beemster *et al.*, 2003.These observations indicated that the RAM shootward border determined in these experiments was located (i) beyond the cell proliferation domain: most of the cells did not divide anymore and only elongated, and (ii) within the transition domain: even if some cells were visibly highly vacuolated, few occurrences of division were observed beyond the shootward border.

### Cell division rate in the RAM

**Fig. 4.**
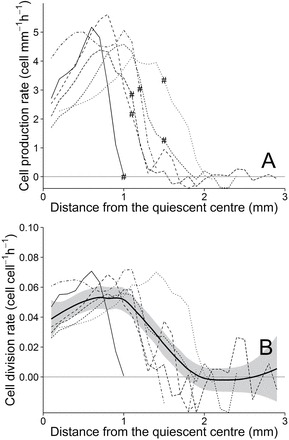
Cell production rate (A) and cell division rate (B) along the root apex determined for control roots. Line types stand for different individuals. Hash signs in A indicate the position of the RAM shootward border. Bold line in B is a smooth curve along the whole population.

Cell production rate, characterizing the local cell production along the root apex, was computed from velocity and cell length profiles assuming steady-state of growth and using a continuum mechanistic formula i.e. the continuity equation (Fig. 4; Erickson and Silk, 1980; Fiorani and Beemster, 2006). To characterize division activity at the cell scale, cell division rate was calculated as the ratio of the cell production rate by the local mean cell density. The high resolution for the particle image velocimetry and the use of a flexible growth equation allowed measuring expansion accurately even in the cell proliferation domain. Moreover, the 500 cells measured per millimetre within the RAM, for a total of 4900 cells measured on six roots, ensured a strong confidence in the cell length profiles, even if it included a smoothing procedure.

For all roots, cell production rate reached a nearly common maximum (4–5.5 cell mm^–1^ h^–1^). For most roots, the RAM shootward border was located in the zone where cell production rate rapidly decreased (Fig. 4A). Cell division rate peaked at around 0.05–0.07 cell cell^–1^ h^–1^ (Fig. 4B). Given the growth rate along the RAM, a cell travelled more than half a millimetre in 15h. In this context, the cell cycle duration, that is the inverse of cell division rate, can only be calculated in the most apical part of the RAM where the growth rate kept relatively constant along such time interval. In the cell proliferation domain, cell cycle duration was about 30–35h for four roots and between 17–20h for the two others. These durations are similar to the ones already reported for *Arabidopsis*, ranging from 18–35h depending on the approach used (Fujie *et al.*, 1993; Baskin *et al.*, 1995; Beemster and Baskin, 1998). They are also close to the ones reported in roots of other species as studied in Grif *et al.* (2002), which is around 15–30h with a large variability depending on distance from the quiescent centre, species, and used method. In herbaceous species, perennials show longer cell cycle duration than annuals (Francis *et al.*, 2008). In the elongation zone, the noise around zero in cell production rate and cell division rate resulted from fewer cells per millimetre owing to their longer length.

Both cell production rate and cell division rate showed similar profiles along the root apex (Fig. 4). Although not being typically bell-shaped, it seems that cell division rate was not constant over the RAM, showing a slight increase from the quiescent centre until a maximum and then decreasing with a steeper slope. The shape of these curves is quite different from the actually supposed steady-state of cell division rate in the RAM, as theoretically suggested (Baskin, 2013). Data clearly sustaining the steadiness of cell division along the RAM are available for *Arabidopsis* (Beemster and Baskin, 1998) and maize roots (Muller *et al.*, 1998). Growth in diameter could result in an underestimate of cell production rate and of subsequent cell division rate. Here, more than 50% of the growth in diameter for cortical cells occurred in the very first 0.1mm after the quiescent centre and 80% in the first 0.3mm. As the apical increase in cell division occurs over 1mm for some roots, it could not be ascribed to a technical bias related to growth in diameter. The heterogeneity among individual profiles of cell division rate suggested that the non-steadiness of division rate was not a bias due to the use of fitting functions. Thus, in accordance with works from Sacks *et al.* (1997) on maize roots, these results strongly support a non-steady activity of cell division within the poplar RAM.

### Impact of the osmotic stress

**Fig. 5.**
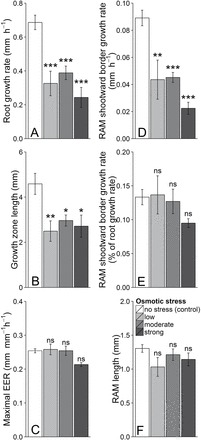
Impact of the osmotic stress: on root growth rate (A), on length of the growth zone (B), on root maximal elemental elongation rate (EER, C), on growth rate at the RAM shootward border (D) which is also given in percentage of root growth rate (E) and on the RAM length (F). Values are means per treatment±standard error. Stars indicate significant differences relative to control (alpha risk: ***≤0.001<**≤0.01<*≤0.05; ns: not significant).

In response to osmotic stress, both root growth rate and length of the growth zone were significantly reduced, by 43–65% for the root growth rate and by 37–46% for the length of the growth zone as compared with controls (Fig. 5A, B). Such responses were already reported under water stress and have been well described especially for roots of maize (Spollen and Sharp, 1991), soybean (Yamaguchi *et al.*, 2010), or pine (Triboulot *et al.*, 1995). Similar results were observed in other organs such as in maize leaves (Tardieu *et al.*, 2000). The different levels of applied osmotic stress (from 0.21–0.37MPa) induced responses of the same intensity. Higher stress levels stopped root growth in these experiments. These responses underlined the strong sensitivity of poplar root growth to osmotic stress. Absence of dose-dependent response to PEG could alternatively indicate PEG toxicity or hypoxia. However, the osmotic stress was applied for a short time (a few hours) and using large PEG molecules (4000g mol^–1^), reducing the risk of PEG toxicity (Janes, 1974), and no change in oxygen level was detected between PEG-added and control solutions. The maximal elemental elongation rate was not affected by osmotic stress (Fig. 5C), indicating that the capacity of cellular expansion was not affected under treatment. This result is somewhat discrepant with commonly reported water stress response patterns (Sharp *et al.*, 1988; Liang *et al.*, 1997) but has already been observed in response to salt stress (West, 2004) and to exogenous application of synthetic cytokinin (Beemster and Baskin, 2000).

Cell expansion results from cell wall yielding that generates the driving force for water uptake (Cosgrove, 1986). Depending on the position along the root apex, it is accompanied by an increase either of the cytosol or of the vacuole. The cytosolic growth occurs in the RAM, ends in the transition zone and corresponds to a slow cell expansion associated with cell division. Vacuolization starts in the transition zone, ends at the end of the growth zone, and is the main factor driving root growth by leading cells that stop dividing to their final size. Here, cell expansion in the RAM was quantified by the velocity at the RAM shootward border which was positioned using the *in vivo* method. Even if a small part of this growth could be due to vacuolization, most of it was cytosolic growth. Growth rate at the RAM shootward border was strongly affected by the osmotic stress with a decrease of 50–80% compared with control (Fig. 5D). This decrease closely paralleled the one of the root growth rate (Fig. 5A), highlighting a general decrease of cell expansion in the whole growth zone, whatever the process involved. Finally, about 10–15% of the root growth rate was due to cytosolic growth in the RAM (Fig. 5E), a non-negligible proportion that should be considered when depicting total root growth rate.

Contrary to the growth zone, the RAM length was unaffected by the osmotic stress (Fig. 5F). In addition, histological analyses revealed that cell size distributions were constant in the RAM whatever the treatment (Supplementary Fig. S6), indicating that the number of dividing cells was not affected under this short-term osmotic stress. In this frame, the cell production by the RAM (that is the cell flux at the RAM shootward border) depends only on cell division rate. Given the decrease of cytosolic growth in response to osmotic stress, the proliferative cells will take more time to reach the threshold above which they achieve mitosis, the division status of a cell being highly correlated with its length (Campilho *et al.*, 2006). These data suggest that cell division rate decreased in response to short-term osmotic stress, which is in line with the high sensitivity of the cell cycle duration to other environmental changes (Grif *et al.*, 2002).

## Concluding remarks

The *in vivo* methodology for RAM length measurement presented here for poplar is consistent with data classically obtained from cell length measurements. The strong relationship found for maize and for *Arabidopsis* sustain that this methodology can be generalized to various species. Kinematic analyses provide new highly resolute spatiotemporal data concerning cell elongation in the growth zone, including the RAM. Coupling these analyses to cell length profiles, these results sustain a non-steady activity of cell production rate and cell division rate along the RAM. From *in vivo* measurements, it was shown that the homeostasis of the RAM length in response to a short-term osmotic stress associated to a lower root growth rate and a shorter growth zone length. However, the response of plant growth to stress generally involves a rapid growth inhibition followed by a recovery and accommodation to the new condition (Skirycz and Inzé, 2010). The timescale of the growth analysis needs to be considered and experiments with longer timescale are required to bridge the gap. Temporal monitoring of the RAM length in mutants or in response to chemicals will improve the understanding of growth acclimation to stresses.

## Supplementary data

Supplementary data are available at *JXB* online

Figure S1. Cell length frequency distribution in the cell proliferation domain.

Figure S2. Example of cell length profile in the first millimetres of a root apex, excluding the root cap.

Figure S3. Longitudinal semi-fine section of a *Populus* root apex coloured with toluidine blue.

Figure S4. Relationship between RAM length obtained from cell length profile and RAM length obtained from the combination of infrared and velocity profiles in maize roots.

Figure S5. Relationship between RAM length obtained from cell length and RAM length obtained from infrared pictures in *Arabidopsis* roots.

Figure S6. Cell length frequency distributions in the proliferation domain of the RAM of control and osmotic stressed roots.

Supplementary Data
