# Supplementary material for: Length and activity of the root apical meristem revealed in vivo by infrared imaging
Source: J Exp Bot. 2014 Dec 24;66(5):1387–95. doi: 10.1093/jxb/eru488 (PMC4339598; doi:10.1093/jxb/eru488)
Supplement: Supplementary Data [file supp_66_5_1387__index.html]

Length and activity of the root apical meristem revealed in vivo by infrared imaging — Length and activity of the root apical meristem revealed in vivo by infrared imaging — Supplementary Data 

# Length and activity of the root apical meristem revealed *in vivo* by infrared imaging

## Supplementary Data

Data files

**Files in this Data Supplement:**

- Supplementary Data - Supplementary Data
