# Supplementary material for: Length and activity of the root apical meristem revealed in vivo by infrared imaging
Source: J Exp Bot. 2014 Dec 24;66(5):1387–95. doi: 10.1093/jxb/eru488 (PMC4339598; doi:10.1093/jxb/eru488)
Supplement: Supplementary Data [file supp_eru488_jexbot134403_file001.pdf]

Manuscript title: Length and activity of the root apical meristem revealed *in vivo* by infrared imaging

Bizet François, Hummel Irène, Bogeat-Triboulot Marie-Béatrice.

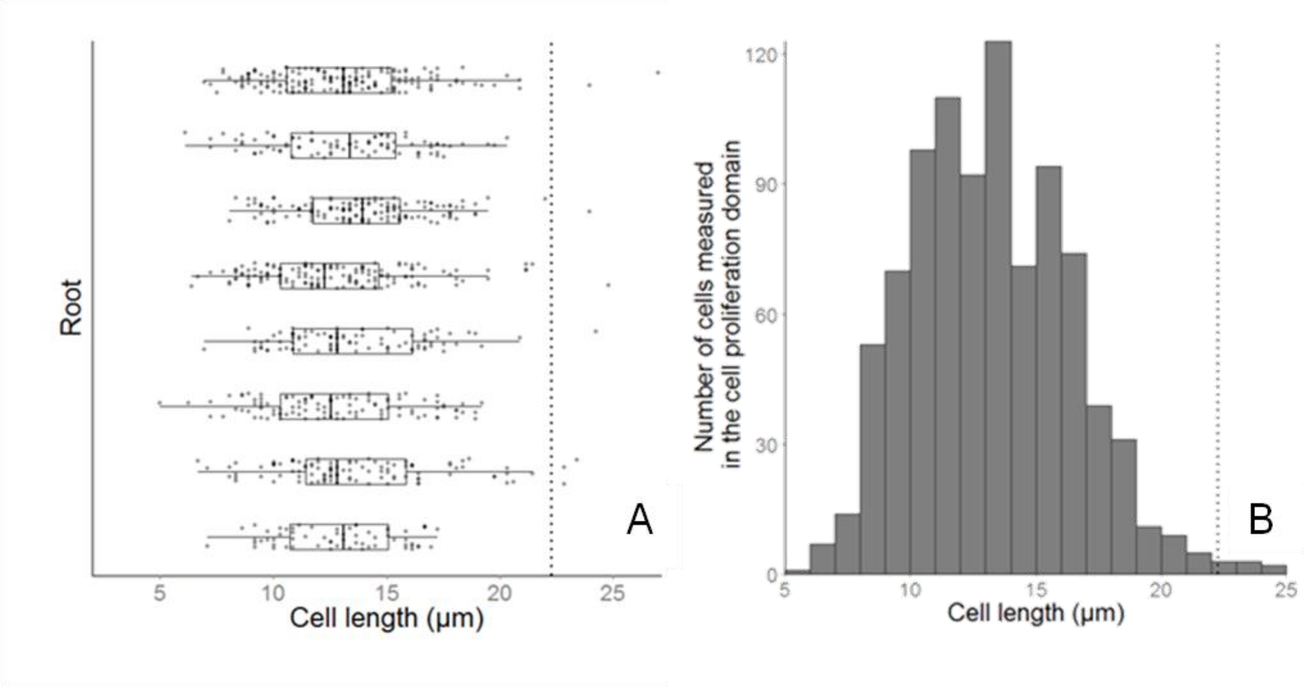

Supplementary Figure S1. Cell length frequency distribution in the cell proliferation domain. A: Individual distribution for eight roots. B: distribution of the merge of these eight roots. Dotted vertical lines indicate the threshold in cell length (set at 80 pixels = 22.3 µm), used to determine the position of the shootward border of the meristem.

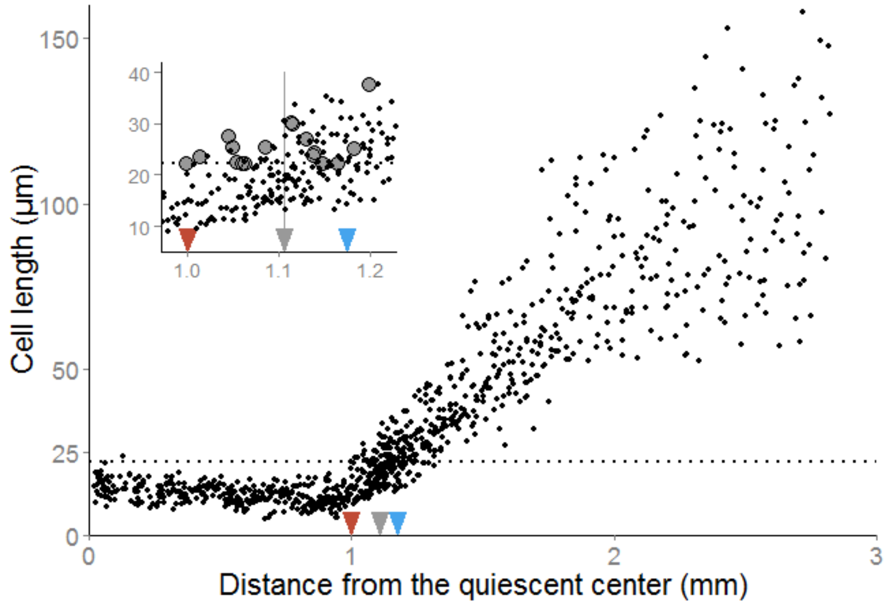

Supplementary Figure S2. Example of cortical cell length profile in the first millimeters of a root apex, excluding the root cap. Dotted line indicates the cell length threshold used to determine the position of the RAM shootward border from cell lengths. Arrows indicate the shootward border positions determined from cell length (in grey), infrared pictures (in red) and velocity profiles (in blue). The subplot displays a zoom in the shootward border of the meristem and grey dots correspond to the first cell of a cortical cell file that was longer than the threshold. Through the combination of infrared pictures and velocity profiles, the shootward border was set at a position which was close to that obtained from direct cell length measurements.

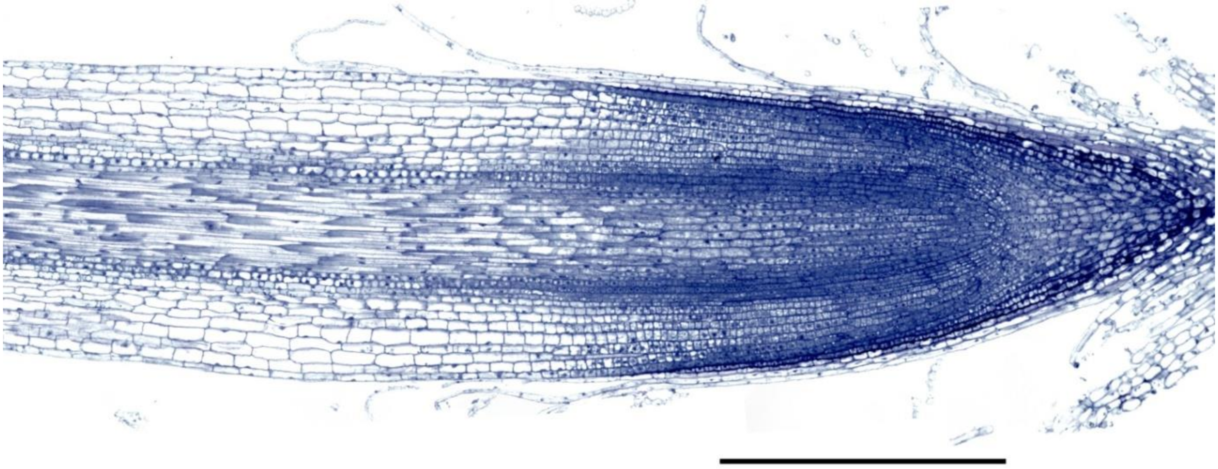

Supplementary Figure S3. Longitudinal semi-fine section of a *Populus* root apex colored with toluidine blue. Scale bar = 0.5 mm.

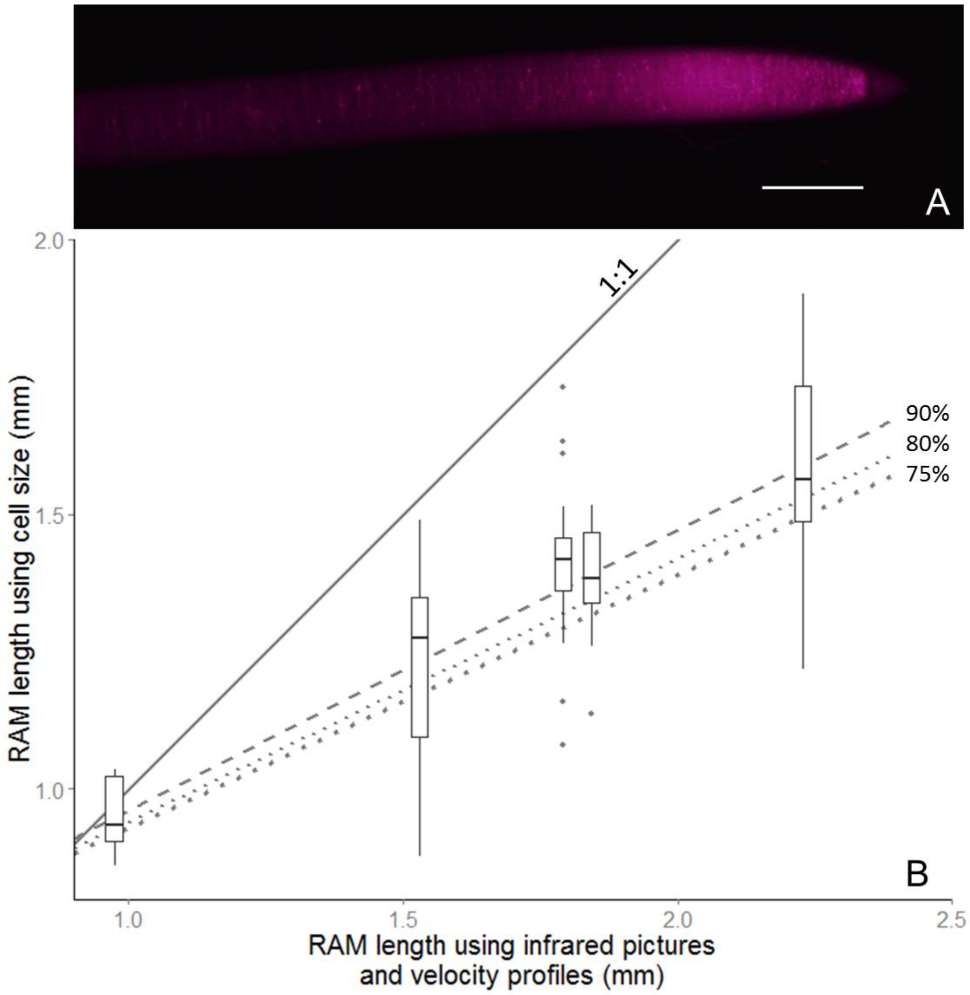

Supplementary Figure S4. RAM length in maize roots. A) Infrared picture of a maize root apex (scale bar = 1 mm). B) Relationship between RAM length obtained from cell length profile and from combination of infrared and velocity profiles, determined for five maize roots. Dashed line indicates linear regression obtained with an infrared threshold of 90% of maximal value (slope = 0.51**; y-intercept = 0.45***; alpha risk: *** ≤ 0.001 < ** ≤ 0.01 < * ≤ 0.05). Dotted lines indicate significant relationship obtained with infrared threshold = 75% or 80%. Solid line indicates 1:1 relationship.

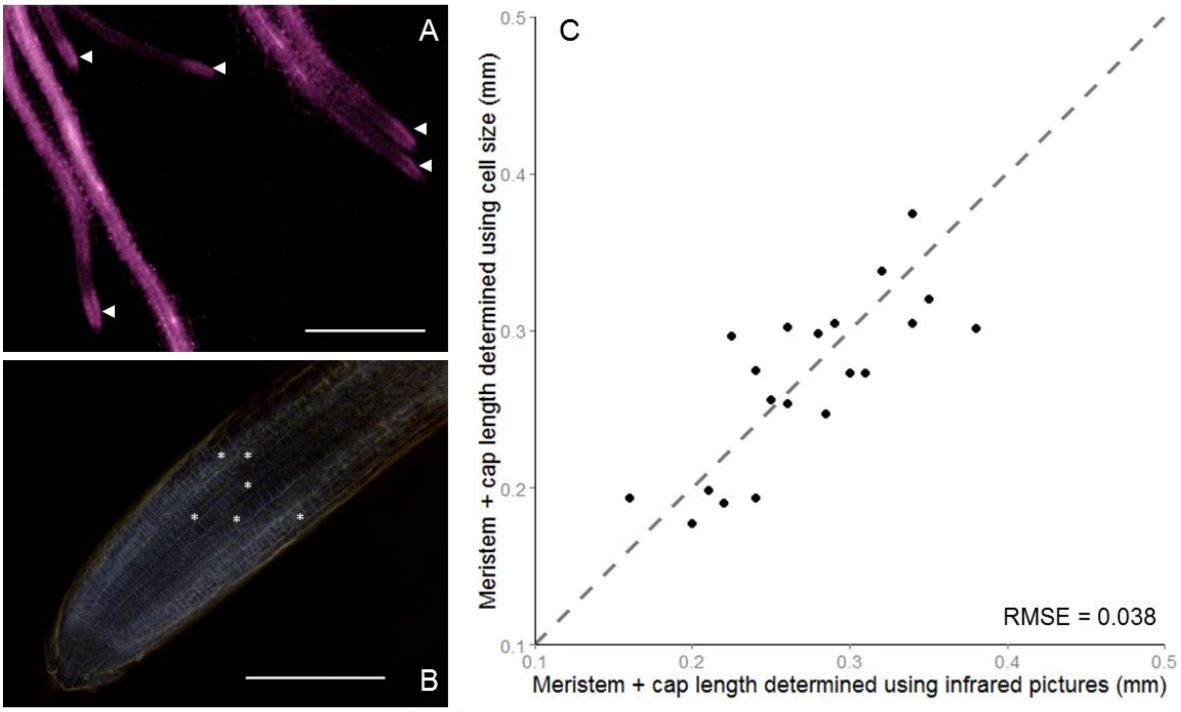

Supplementary Figure S5. RAM length in Arabidopsis thaliana. A) Infrared picture of an Arabidopsis root system showing several apices with high pixel intensity (white arrows; scale bar = 0.5 mm). B) Arabidopsis root viewed under light microscopy with a one hundred magnification after fixation and clearing in a chloral hydrate solution for 4 hours (asterisk denotes the first rapidly elongating cells in each cell file, indicating the end of the RAM; scale bar = 0.2 mm). C) Relationship between (RAM + root cap) length obtained from cell length and (RAM + root cap) length obtained from infrared pictures in twenty roots of Arabidopsis (Col-0) plants grown in Petri dishes on MS-agar medium (16h light period, 17°C/22°C, night/day). The root cap was included since the resolution on our digital camera prevented the exact location of the quiescent center on infrared pictures. Using higher magnification and an experimental device dedicated to Arabidopsis would improve the IR-based estimation of the RAM length.

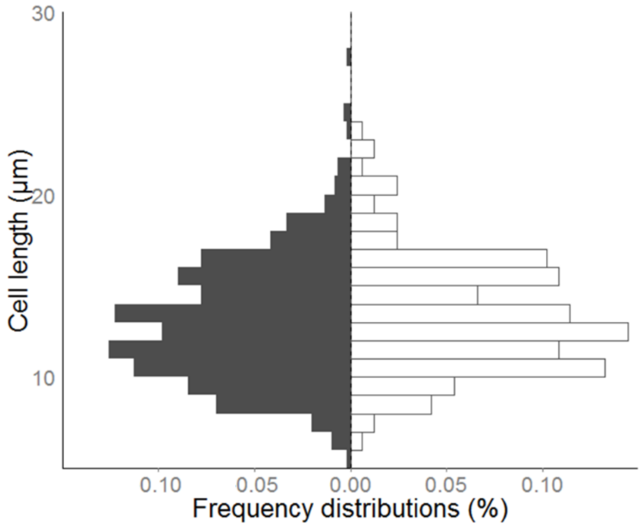

Supplementary Figure S6. Cell length frequency distributions in the proliferation domain of the root apical meristem for control (white bars) and osmotic stress (dark bars).
